# The Harvard medical practice study trigger system performance in deceased patients

**DOI:** 10.1186/s12913-018-3839-6

**Published:** 2019-01-08

**Authors:** Dorthe O. Klein, Roger J. M. W. Rennenberg, Richard P. Koopmans, Martin H. Prins

**Affiliations:** 1 Department of Clinical Epidemiology and Medical Technology Assessment (KEMTA), Maastricht University Medical Centre+, Maastricht, the Netherlands; 2 Department of Internal Medicine, Maastricht University Medical Centre+, Maastricht, the Netherlands; 3 Department of Epidemiology, School for Public Health and Primary Care, Maastricht University, Maastricht, the Netherlands

**Keywords:** Medical record review, Reproducibility, Kappa, PABAK, Triggers, Hospital

## Abstract

**Background:**

To detect possible threats to quality and safety, multiple systems have been developed. One of them is retrospective chart review. A team of experts scrutinizes medical records, selected by trigger systems, to detect possible adverse events (AEs). The most important AEs and more hints for possible improvement of care appear in deceased patients. Using triggers in a sample of these patients might increase the performance and lower the burden of scrutinizing records without possible preventable AEs. The aim of this study was therefore to determine the performance of the trigger system in a sample of deceased patients and to calculate the specificity and the sensitivity of this trigger system for predicting AEs.

**Methods:**

We performed a study in which the records of deceased patients were screened for triggers by a team of trained nurses. A sample of 100 medical records was randomly selected out of records which had been screened between 2012 and 2015 for the first time, prior to the study in 2016. For the determination of significant differences between the first and second screening, McNemar’s test of symmetry was used. Also, observed agreement, Cohen’s Kappa and prevalence-adjusted and-bias-adjusted-kappa (PABAK) statistics were calculated. This was done for the two trigger rounds on both any trigger present and for every trigger separately.

**Results:**

The observed agreement for any given trigger was 75% with a Kappa and PABAK of 0.5. For the individual triggers, the observed agreement was on average 90%. The corresponding Kappa was on average 0.42 (range: − 0.03-0.78) and the average PABAK was 0.8 (range: 0.44–0.92). Two adverse events were found in cases without triggers previously. The recalculated specificity and sensitivity for the original population were 58 and 92% respectively.

**Conclusions:**

For the reproducibility of triggers it seems that some perform better than others, but on average this is to our opinion suboptimal. The low specificity implies that many records are selected without AEs. This leads to a high false-positive rate making this labour-intensive record review process costly. Therefore, research for better and more expedient systems is required.

## Background

Improving quality and safety of care in hospitals has become an important focus of health care policy in the past decades. This was initiated by reports such as “to err is Human” (1999), and in the Netherlands by the report “adverse events in Dutch hospitals” (2004) [1, 2]. The latter study was repeated in 2008 and 2012. Although there was an improvement, still a considerable number of (potentially preventable) adverse events (AEs) was found [3, 4]. Also a report by Landrigan et al. (2010) stated that further efforts are necessary to improve safety strategies and to monitor health care safety over time [5]. To detect possible threats to quality and safety, multiple systems have been developed. One of them is retrospective chart review. A team of experts scrutinizes medical records to detect possible AEs. The involved departments should then be able to learn from these events and improve their care by increasing awareness and adapting protocols or guidelines.

It is clear that screening of the medical records of all patients by specialists is time-consuming and costly [6]. Therefore, trigger systems have been developed to select cases in which an AE is probably present [7]. Triggers are clues which alert screeners for potential AEs (for example “unplanned transfer to the intensive care unit”). The medical record can then be thoroughly reviewed to determine if an actual AE has occurred. There are two main trigger systems used widely and the triggers are usually applied to the medical files by trained screeners in both systems. The first one was developed for the Harvard Medical Practice Study (HMPS) study and has 18 triggers [8]. Thereafter, the Institute for Healthcare Improvement (IHI) tried to improve the performance of this trigger tool and developed their system with 54 triggers [9]. Both systems are used for retrospective medical record review. However, in contrast to the IHI trigger system, the HMPS method is mostly used for research purposes [10]. Although the HMPS trigger set is rather old, it is still used in national screening programs to evaluate patient safety in hospitals [10–13].

Usually, these trigger systems are applied to records of discharged patients which are functioning well. However, the literature shows that the most important AEs and more hints for possible improvement of care in all patients appear in deceased patients [14–17]. Moreover, several studies have shown higher numbers of preventability of AEs in this subgroup [2, 12, 14, 18]. Thus, apart from optimizing the trigger tool itself, using it in this sample of patients might increase the performance and lower the burden of scrutinizing records without possible preventable AEs [19]. Finding AEs and possible points of improvement is considered important by our hospital and therefore medical record review of deceased patients has been applied for many years.

We wondered if the performance of the trigger system would be better in a sample of deceased patients because it was shown that in these patients more triggers can be easily found [20]. Therefore, we performed a study in which the records of deceased patients were screened twice for these triggers by the same team of trained nurses. Our aim was to determine the agreement for the two trigger rounds on both any trigger present and for every trigger separately using the HMPS trigger system in deceased patients.

## Methods

### Medical record review

#### Screeners

Since 2008 a stable team of seven nurses with broad clinical experience (all working more than 10 years in their clinical department) has screened the medical records of all deceased patients for the presence of triggers from the HMPS system. They were trained in record review and patient safety initially for the national monitoring on triggers and AEs as described by the Nederlands Instituut Voor EersteLijnsgezondheidszorg (NIVEL) [21]. During their work at the intensive care and emergency departments, they have been confronted frequently with dying patients. This has resulted in them being pre-eminently suitable to judge events concerning these patients.

#### Trigger system

**Fig. 1:**
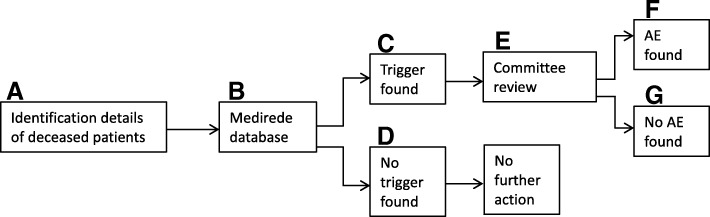
Procedure of medical record review in our centre. (**a**) Identification details of all deceased patients are inserted in the Medirede® database (**b**) and the corresponding medical record is screened by one of the nurses. (**c**) When one or more triggers are found in the medical record (registered in Medirede), the case is flagged and will continue to the review committee. (**d**) When no trigger is found (also registered in Medirede), no further action will be taken in the normal screening procedure. (**e**) The committee evaluates the cases and will determine whether (**f**) an adverse event occurred or (**g**) not.

Patient identification details of those who died during their stay were uploaded in a secured software program designed to aid medical record review of deceased patients (Medirede®, Clinical File Search version 3; Mediround BV, 2015). Then the matching records were screened by one of the nurses from the team using the HPMS trigger system. If a medical record contained at least one trigger, it was regarded as positive and was then forwarded to the review committee and scrutinized for the potential presence of AEs. This is shown in Fig. 1.

We used a slightly adapted version of the HMPS trigger system to make it suitable for the screening of deceased patients [22]. The triggers regarding transfer to another acute care hospital and unplanned inappropriate discharge to home were omitted as they have no relevance in deceased patients. The same triggers were used throughout the whole period except the trigger regarding readmission of the patient which was changed in 2013 (originally; the patient was admitted before (< 12 months) for a reason related to index admission). Analysis of our database showed that this trigger was not discriminative for (potentially preventable) AEs (data not shown). For example: within the 12-month period, many patients were selected with planned repeated chemotherapy or planned reversal operations (which by their very nature are not related to AEs and thus not useful for our purposes). Therefore, the definition of trigger 1 was adapted to “patient has been admitted in the previous *three months* for a reason related to the index admission”. Example of cases with corresponding triggers is explained in further detail elsewhere [23]. Each record was reviewed twice by one of the nurses, once prior to the study in the context of their regular work as screeners and once during this study.

#### Review committee

**Fig. 2:**
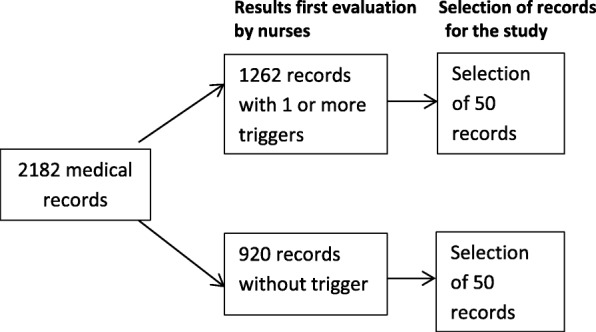
Study flow

Usually, only medical records with a trigger were forwarded to one of the members of the review committee. The committee consisted of several clinical specialists representing the departments with most of the in-hospital deaths who were trained to identify AEs according to NIVEL standards [21]. For this study, they also evaluated records without a trigger (Fig. 2, D). After an evaluation of the medical record, they decided together (in a consensus meeting) whether an AE had occurred during the hospitalisation of the patient.

### Study

This study was performed in 2016 at the Maastricht University Medical Centre (MUMC+), a large teaching hospital in the south of the Netherlands. The medical records that were used in this study included a sample of all inpatient deaths between January 2012 and January 2015 (in total 2182 cases). The study protocol was approved by the medical ethics committee of our hospital.

### Data and analysis

We aimed to get a point estimate with an exact 95% confidence level and a confidence interval of 5% to each side, based on the total number of records. Hence, we needed to include at least 92 cases. Therefore, a sample of 100 medical records was randomly selected (with the use of Excel’s random generator) out of records which had been screened in the preceding years. Characteristics of the patient sample are presented in Table 1.

**Table 1:** General characteristics of the patient sample (2012–2015)

Average age (years)	67.6
Gender	52% male
Admission specialism	5% paediatrics 7% other 9% neurology 12% lung diseases 14% ICU 14% cardiology 15% surgery 24% internal medicine
Average length of stay (days)	13.5

We selected fifty of these records from the set without triggers in the first screening and fifty from the set with at least one trigger present. The study flow is depicted in Fig. 2. To ensure that the nurses were blinded to the results of the first screening we changed the ID numbers of the records, making it impossible to consult previous results.

In the primary analysis, the nurses were analysed as a group instead of as individuals. If a small subsample of at least ten cases was triggered by the same nurse during the first and second round, we calculated the Kappa also separately in this subgroup analysis.

For the determination of significant differences between the two screening rounds, McNemar’s test of symmetry was performed. Observed agreement and Cohen’s Kappa statistics (with 95% confidence interval; CI) were calculated between the two trigger rounds on both any trigger present and for every trigger separately. For the calculation of the observed agreement (reliability), we divided the total number of cases with a comparable judgment in both screening rounds by the total number of reanalysed records (100). We also checked whether there was a difference between objective triggers (trigger 1, 3, 4, 5, 6, 7, 8, 9, 10, 11 and 14) and subjective triggers (trigger 2, 12, 13, 15).

Furthermore, we calculated prevalence-adjusted and bias-adjusted kappa (PABAK) and reported this along with Kappa, to show how data would have been with equal distributions of positive and negative test results. Finally, we determined prevalence-indexes and bias-indexes [24].

Analyses were carried out using IBM SPSS Statistics version 23 (IBM Corporation, 2015). A *p* < 0.05 was indicated as significant.

All selected records were evaluated by the review committee (regardless if triggers were found by our screeners). We recalculated the numbers of triggers and AEs to represent the original number of patients in the specific population. With this information, we were able to calculate the specificity and sensitivity of the trigger system.

## Study results

### Results for any trigger present

Table 2 shows that the second screening revealed 20 new cases with a trigger. 45 cases had a trigger in both screening rounds. This resulted in 65 records with a trigger in this study. After the screening in the study, 35 records didn’t have a trigger. 30 of these records didn’t have a trigger in both screening rounds. 5 of these records had a trigger after the first screening but remained without trigger after the screening in the study.

**Table 2:** Numbers of triggers and AEs in the first and second round

Trigger first round	Trigger second round (study)	Number of cases	AE first round	AE second round (study)
+	–	5	0	0
+	+	45	25	28
–	+	20	NA^a^	5
–	–	30	NA^a^	2

^a^NA: not assessed; because there was no trigger in the first round, these records were not investigated in the first round

The observed agreement for the triggers was 75%, and the corresponding Cohen’s Kappa was 0.50 (95%CI 0.34–0.66). PABAK was calculated as 0.5 (95%CI 0.29–0.66). An exact McNemar’s test confirmed that there was a significant difference in the proportion of positively triggered records the first and second time, with *p* = 0.004.

### AEs found in relation to trigger status

In the 20 cases with a newly detected trigger, 5 AEs were found. In the cases without a trigger in both rounds 2 AEs, were found. After recalculating the numbers of these proportions to represent the whole population we found a specificity of 58% (95% CI 55.7–60.8) and a sensitivity of 92% (95% CI 90.1–94.2) for detecting AEs.

### Results for the individual triggers

In Table 3, the results are shown for the individual triggers during the first and second screening. Trigger 12 was not present in both screening rounds. Eleven triggers were more often detected during the assessment in this study. Kappa for agreement ranged between − 0.03 and 0.78 for the individual triggers, with an average of 0.42. The average PABAK was 0.80. The observed agreement was on average 90%.

**Table 3:** Triggers given the first and second time of screening

Triggers	Description	First time positive (N)^1^	Second time positive (N)^a^	Percentage agreement (%)	Kappa agreement (95%CI)	McNemar’s test (p-value)	PABAK^b^ (95%CI)	Bias index	Prevalence index
Trigger 1	Unplanned readmission^c^ (within 3 months) after discharge from index admission	13	17	88	0.53 (0.30–0.76)	0.39	0.76 (0.58–0.88)	− 0.04	− 0.7
Trigger 2	Hospital-incurred patient injury (temporarily or lasting)	8	4	90	0.12 (−0.17–0.41)	0.34	0.80 (0.63–0.90)	0.04	−0.88
Trigger 3	Adverse drug reaction	2	6	92	−0.03 (− 0.06–0.002)	0.29	0.84 (0.68–0.93)	− 0.04	− 0.92
Trigger 4	Unplanned transfer to the ICU	15	18	93	0.75 (0.57–0.92)	0.45	0.86 (0.70–0.94)	−0.03	− 0.67
Trigger 5	Unplanned return to the operating room	8	12	96	0.78 (0.59–0.99)	0.13	0.92 (0.78–0.98)	−0.04	− 0.80
Trigger 6	Unplanned removal, injury or repair of an organ during surgery	5	6	95	0.52 (0.15–0.89)	1.00	0.90 (0.75–0.97)	−0.01	−0.89
Trigger 7	Healthcare related infection or sepsis	20	22	88	0.64 (0.45–0.83)	0.77	0.76 (0.58–0.88)	−0.02	− 0.58
Trigger 8	Other complications such as CVA/lung embolism/acute myocardial infarction/TIA	17	16	89	0.60 (0.38–0.81)	1.00	0.78 (0.60–0.89)	0.01	−0.67
Trigger 9	Development of neurological deficit which was not present on admission	5	7	92	0.29 (−0.06–0.65)	0.73	0.84 (0.68–0.93)	−0.02	− 0.88
Trigger 10	(Initial) unexpected and/or sudden death, (no palliative care)	12	14	84	0.29 (0.03–0.55)	0.80	0.68 (0.49–0.81)	−0.02	−0.74
Trigger 11	Cardiac or respiratory arrest	12	11	93	0.66 (0.42–0.89)	1.00	0.86 (0.70–0.94)	0.01	−0.77
Trigger 12^d^	Injury related to abortion or delivery	–	–	–	–	–	–	–	–
Trigger 13	Dissatisfaction with care documented in the record	2	5	95	0.27 (−0.17–0.71)	0.38	0.90 (0.75–0.97)	−0.03	− 0.93
Trigger 14	Documentation indicating a legal claim or complaint procedure^e^	0	2	–	–	–	–	–	–
Trigger 15	Other patient complication	5	27	72	0.04 (−0.10–0.189)	0.00	0.44 (0.23–0.62)	−0.22	− 0.68

^a^Negative is 100-N

^b^PABAK: prevalence and bias adjusted kappa

^c^A readmission was considered as unplanned if admission was through the emergency department

^d^This trigger was not found in both rounds of the medical record analysis

^e^No statistics could be computed because trigger 14 is not present in the first round

McNemar’s test was executed for every single trigger but was only significant for trigger 15 (*p* < 0.0001). Trigger 15 was significantly more found in the second round. Furthermore, the objective triggers had a higher average Kappa (0.46) compared to the subjective triggers (average K = 0.28). The corresponding PABAK was 0.82 and 0.71, respectively.

### Subanalyses

These subanalyses were executed because twelve cases were analysed by the same nurse during the two screenings. For any trigger present, the Kappa was 0.63 (95%CI 0.15–1), PABAK was 0.67 (95%CI 0.25–1). The average Kappa of the individual triggers was 0.70 and the average PABAK was 0.83.

If these twelve cases would be excluded from the total analyses, the Kappa for any trigger present would be 0.48 (95%CI 0.30–0.67) (compared to 0.5 with these 12 included) and PABAK would be 0.48 (95%CI 0.29–0.66). The average Kappa for the individual triggers would be 0.39 (compared to 0.42 in the total sample) and corresponding PABAK 0.78.

## Discussion

In this study, we have shown that the reproducibility (Kappa) of the presence of any trigger present in deceased patients in the hospital was 0.5 (95%CI 0.34–0.66). The average Kappa of individual triggers was 0.42 (range 0–0.78). Our average Kappa of 0.5 (moderate agreement according to Landis et al), appears to be slightly lower than results found in other studies, where a range between 0.49–0.76 was reported [3, 4, 21, 25–28]. However, compared to three Dutch reports which included results of screening for triggers in a sample of cases in 21 hospitals, our Kappa was in the same range [3, 4, 21]. Naessens (2010) and Ock (2015) evaluated the inter-rater reliability for individual triggers selected either from the HMPS study or the IHI trigger system or both. Four of the triggers investigated by Naessens et al., were comparable to our triggers. Half of these had a higher Kappa agreement in our study compared to Naessens et al. Two out of the three comparable triggers in the study of Ock et al. (2015) performed better in their study compared to ours [29, 30]. However, again, the population here was sampled from living non-pediatric inpatients.

Concerning the average observed agreement for individual triggers, Unbeck et al. (2014) reported that the reproducibility of the individual triggers was on average 46%, in comparison with a 67% reproducibility in our study. The total agreement for any trigger present was 65.0% compared to 90% in our study [31]. Regrettably, Unbeck et al. didn’t report the performance of the triggers on an individual level and studied only living pediatric inpatients. Therefore, this is the first study investigating the performance of the individual triggers of the HMPS trigger system solely in deceased patients. Not surprisingly, objective triggers were more reproducible than subjective triggers.

The Kappa coefficient is influenced by the prevalence of the condition and by bias. Therefore, we also calculated the PABAK. This improved the reliability score, resulting in moderate substantial to almost perfect inter-tester reliability for the individual triggers. An exception was the trigger concerning other patient complications which showed almost no improvement with an end result still well below moderate reliability. The outcomes of these calculations suggest that the low value of Kappa was influenced mainly by the low prevalence of triggers.

Obviously, the performance of the trigger system is important. It should not miss records with serious and potentially preventable AEs and preferably not select any records without AEs. Because trigger systems are used as an aid to reduce the burden of scrutinizing all records, it implies that important AEs could be missed. The fact that new cases with triggers were found in the second round supports this idea.

Due to our random sample of records, we believe that the calculation of the estimated sensitivity and specificity approaches reality. Therefore, when we apply these values found in this study to the entire population of deceased patients in our hospital the false negative rate would be 8%.

The high sensitivity of the system to find cases with an AE was rather comforting. In contrast to a high sensitivity, the specificity of this trigger system was rather low (58%) compared to most of the other studies [22, 32–35]. This results in a substantial number of cases that have to be scrutinized without finding an AE. However, equal results were presented by Howard et al. (2017) and our results were slightly better in comparison to Neubert et al. (2006), Eggleton et al. (2014) and Matlow et al. (2011) [36–39].

The variability in triggered cases with a low Kappa suggests unfavourable characteristics of this system. This possibly results in considerable useless time-consuming scrutinizing of records by expensive specialists. Solutions to improve efficacy could be the use of more reproducible triggers (such as the objective ones), combining triggers with patient characteristics, or fully computerized trigger detection by “data mining” software [23]. Before implementing such adaptations, we suggest thorough research concerning the exact performance and costs for finding preventable AEs. However, at the moment there are no better systems available for case selection.

Among the strengths of our study is the fact that the nurses were blinded to the results of the first trigger round. Furthermore, in our system, there were no time limitations while searching for triggers. We, therefore, assume that cases were investigated thoroughly and complete which makes the possibility of a missed trigger as low as possible.

A disadvantage of our study is the small randomly selected sample of all cases that were screened previously. However, this sample was strong enough to detect the real proportion of triggers. Another issue could be the selection of deceased patients. Some studies report that a focus on deaths may not be the most efficient approach or an unsuitable indicator to compare the quality of hospitals [11, 40]. Yet, mortality is the event caretakers and patients want to prevent. Of course, departments with low mortality or those who treat non-life-threatening diseases, such as ENT and dermatology, will rarely hear about their AEs from this type of medical record review. As several studies show, AEs don’t have to result in death [2, 26, 41]. They can also cause temporary or permanent injury. Triggers are indications for all AEs, not necessarily for those who cause death. Hence, another chart review system could be more applicable to those departments. Finally, trigger 1 was changed during the time course in which we selected cases for this study. This could have potentially influenced the results. However, trigger 1 was found more often in the second screening round where we expected less often because we shortened the time period making it positive. Therefore, we do not think this influenced our results materially.

Interestingly, more triggers were found during the second round, especially trigger 15 was significantly more present being responsible for most of the difference. In our opinion, supported by a *p*-value < 0.0001 resulting from the McNemar’s chi-square test, this cannot be attributed to chance alone. We suspect that extra attention among the nurses due to the fact that the second round of review was part of a study might have contributed to this. Furthermore, one could suspect an increase in experience although our team of nurses was deployed for many years in a stable team and cases were selected from a recent period. However, some of the cases that were triggered the first time were not found in the second round. Although memorizing the results in a specific case could have given rise to bias, we found only 12 cases that were checked by the same nurse. Excluding these 12 cases did not influence the results significantly.

We realise that we only analysed a small part of the complete process of looking back at our proceedings, determine essential parts, develop new solutions and applying them in future care. Moreover, there is no information about the performance of this trigger system in improving health care. However, we think it is important to increase knowledge about these components to optimise care in the end.

## Conclusion

In conclusion, applying the adjusted HMPS trigger system as an aid to select records of deceased patients with possible AEs has, in our opinion, a suboptimal Kappa possibly influenced by the low prevalence of individual triggers. Awaiting better selection systems this is, however, the best way to avoid doing a time consuming and costly analysis of all cases. Moreover, it can identify possible threats to quality and safety which can then be further investigated by other methods. However, we have to realize that selection of cases for a more thorough investigation by these common trigger systems, with many subjective triggers, is only moderately reproducible with a low specificity for AEs. Therefore, studies to evaluate possible improvements of these systems or even other systems are important to increase the expediency of these costly tools.

## Abbreviations

AE: Adverse event
CI: Confidence interval
HMPS: Harvard Medical Practice Study
IHI: Institute for Healthcare Improvement
MUMC+: Maastricht University Medical Centre
NIVEL: Nederlands Instituut Voor EersteLijnsgezondheidszorg
